# DBS‐Based Hydrophobic Nonionic Supramolecular Eutectogels: Bridging Molecular Insights to Water Remediation

**DOI:** 10.1002/open.70323

**Published:** 2026-10-23

**Authors:** Chiara Carotti, Laura Riva, Alberto Mannu, Arianna Rossetti, Gloria Nicastro, Francesco Briatico Vangosa, Andrea Mele, Maria Enrica Di Pietro

**Affiliations:** ^1^ Department of Chemistry, Materials and Chemical Engineering “G. Natta” Politecnico di Milano Milan Italy; ^2^ INSTM—Local Unit c/o Politecnico di Milano Milano Italy

**Keywords:** bisphenol A, eutectic solvents, HRMAS, rheology, soft matter

## Abstract

Supramolecular hydrophobic eutectogels (HEG) are an emerging class of soft materials. They combine the high solvation ability and sustainability profile of hydrophobic eutectic solvents (HES) with the mechanical stability and easy separation and handling of supramolecular gels. However, the relationship between the molecular organization of the HES liquid phase confined within the supramolecular gel, the bulk properties of the gel matrix, and the functional performance of the final material remains largely unexplored. Here, HEG were prepared by self‐assembly of the low‐molecular‐weight gelator 1,3:2,4‐dibenzylidene‐D‐sorbitol (DBS) at two concentrations (2 and 5 wt%) in four nonionic HES (trioctylphosphineoxide:DL‐menthol, trioctylphosphineoxide:dodecanoic acid, DL‐menthol:oleic acid, and octanoic acid:dodecanoic acid). The HEG were investigated by a combination of rheology, high‐resolution magic‐angle spinning (HRMAS) NMR spectroscopy, and diffusion measurements, providing a comprehensive description of the materials covering mechanical properties, molecular organization, and translational mobility under confinement. Their potential as economically and environmentally sustainable sorbents in water remediation was demonstrated in the removal of bisphenol A (BPA) from aqueous solutions. The extraction efficiency and cross‐contamination arising from HEG component leaching were shown to depend on the interplay between HES‐specific intermolecular interactions and DBS‐induced confinement. Indeed, the supramolecular organization induced by the gelator introduces an additional level of control over molecular mobility, thus affecting both BPA uptake and leaching. Among the investigated systems, trioctylphosphineoxide‐based HEG provided the most favorable balance between BPA removal and leaching of the HEG components, while maintaining their structural and molecular properties after extraction. BPA extraction efficiencies above 98.5% were achieved for the systems containing 5 wt% DBS after 16 h, while quantitative NMR analysis showed low leaching of HEG components (<0.3 wt%). These findings support HEG as versatile materials where molecular organization and mobility, mechanical properties, and pollutant affinity can be tailored to design functional soft materials for separation processes and beyond.

## Graphical abstract

Hydrophobic eutectogels exploit supramolecular confinement to tune molecular mobility, mechanics, and sorption. By correlating these features with extraction performance, this work identifies TOPO‐based eutectogels as the most effective sorbents, achieving efficient bisphenol A removal while minimizing component leaching.

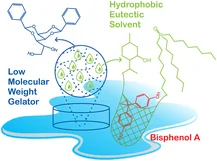

## 1 Introduction

Eutectogels, gels comprising eutectic solvents (ES) as one of the component, are emerging as a versatile family of soft materials [1, 2, 3, 4, 5]. These hybrid systems integrate the adaptable chemistry of liquid ES with the structural robustness of a gel network, leading to materials with adjustable polarity, surface properties, and responsiveness. Among the wide portfolio of possible formulations, supramolecular eutectogels, obtained through the self‐assembly of low‐molecular‐weight gelators (LMWGs), are particularly attractive [6]. Their network is stabilized by noncovalent interactions, such as hydrogen bonding, π–π stacking, and van der Waals forces, endowing them with thermoreversibility, ease of preparation, and often self‐healing and stimuli‐responsive behavior. These features make supramolecular eutectogels highly adaptable and inherently reusable, aligning with the principles of green chemistry and the circular economy. Despite being a recent approach, supramolecular eutectogels have already demonstrated their potential for applications such as drug delivery systems [7, 8], energy technology [9], and wastewater treatment [10].

Most supramolecular eutectogels reported to date are based on ionic ES, mainly comprising choline chloride as the hydrogen bond acceptor and small organic acids (e.g., formic, oxalic, and citric acid) or alcohols (e.g., ethylene glycol, glycerol, xylitol, sorbitol) as the hydrogen bond donor [8, 9, 10]. By comparison, considerably less attention has been devoted to eutectogels prepared from hydrophobic nonionic ES (HES). In these systems, the absence of long‐range Coulombic interactions makes the supramolecular organization predominantly governed by hydrogen bonding and dispersive interactions. Some of us have recently demonstrated that stable supramolecular hydrophobic eutectogels (HEG) can be formed within minutes by self‐assembly of 1,3:2,4‐dibenzylidene‐D‐sorbitol (DBS), a low‐cost commercially available LMWG [9, 11, 12], in several HES based on molecular components (type V HES) [13, 14]. However, little is known about their molecular organization after gelation and its relationship with macroscopic properties, and the understanding of the changes in molecular and bulk properties undergone during application in the presence of additional molecules remained unexplored in previous studies. Understanding this structure–property relationship is essential for the rational design of next‐generation soft materials, particularly when their performance depends on selective interactions with external molecules. To specifically address this knowledge gap, the present work expands the understanding of this emerging class of materials by exploring, for the first time, their potential application as sorbents for water purification. Supramolecular hydrogels obtained by self‐assembly of DBS‐hydrazide derivatives in water have already been used in the extraction of dyes and precious metal ions from water [15, 16]. Beyond demonstrating the use of hydrophobic DBS‐based supramolecular gels as alternative DBS‐pollutant extractants, the novelty of the present work lies in establishing relationships among HES‐specific intermolecular interactions, confinement induced by the supramolecular DBS network, macroscopic mechanical properties, and extraction and leaching behavior. More specifically, the questions we intend to address here are whether (i) supramolecular eutectogels formed in a one‐pot step from commercially available DBS in a set of straightforward HES have potential as sustainable sorbents for water treatment and (ii) a connection between HES‐specific intermolecular interactions and pollutant extraction performance can be established.

The relevance of these questions is underscored by the growing challenge of water contamination. Ensuring access to clean and safe water is indeed one of the major global challenges of the 21st century, stated as Goal 6 of the United Nations Sustainable Development Goals [17]. The increasing occurrence of contaminants of emerging concern (CECs), also termed micropollutants, such as pharmaceuticals, personal care products, dyes, and many other anthropogenic chemicals, has raised significant environmental and health concerns [18]. Current wastewater treatment plants are not specifically designed to eliminate micropollutants; hence, many of these compounds may end up in the aquatic environment, becoming threats to wildlife and spelling trouble for the drinking water industry. Adsorption is a well‐established water treatment technology, but conventional sorbent materials such as activated carbons, clays, zeolites, and metal nanoparticles may suffer from limited selectivity, poor biodegradability, significant environmental toxicity, and/or incomplete removal of target molecules [18, 19, 20]. These limitations justify a continuous interest in the search for innovative, sustainable materials capable of providing efficient, selective, and recyclable removal of organic pollutants from water systems.

Against this background, we selected bisphenol A (BPA) as a model contaminant to evaluate the sorption performance of DBS‐based supramolecular HEG. While being one of the highest production and consumption volume chemicals in the world, BPA is a known endocrine disruptor, and its wide presence in wastewater poses a clear threat to both human health and the environment [21]. While regulations exist for its presence in surface and drinking water [22, 23], uniform wastewater limits have not been implemented across all countries. A previous screening of type V HES, prepared as binary mixtures of terpenes (camphor, carvone, menthol, thymol), carboxylic acids (hexanoic, octanoic, decanoic, dodecanoic, and oleic acids), and a phosphine oxide (trioctylphosphine oxide), demonstrated their high potential for BPA extraction from model aqueous samples, with extraction efficiencies above 97.5% [24]. However, challenges in terms of HES leaching into the water phase emerged, and the sustainability profile of the extraction protocol would benefit from a reduction of the operating steps. To address these limitations, we designed and applied here supramolecular HEG for BPA sorption from water, and we combined spectroscopic and rheological methods to bridge molecular insights, mechanical properties, and extraction indicators, both in terms of BPA extraction and cross‐contamination arising from HEG component leaching. This joint molecular and application‐oriented perspective provides a foundation for the rational development of supramolecular HEG with potential applications beyond water remediation.

## 2 Experimental Section

### 2.1 Materials

The compounds used in this work are summarized in Table S1 and were all used as received without further purification. Structures are shown in Figure 1.

**FIGURE 1:**
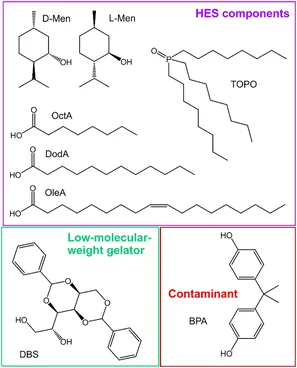
Molecular structures of the HES components used in this work (DL‐menthol, racemic mixture of D‐menthol and L‐‍menthol, Men; trioctylphosphineoxide, TOPO; octanoic acid, OctA; dodecanoic acid, DodA; oleic acid, OleA), the low‐molecular‐weight gelator (1,3:2,4‐dibenzylidene‐D‐sorbitol, DBS), and the target contaminant (bisphenol A, BPA).

### 2.2 Preparation of Hydrophobic Eutectic Solvents (HES)

Four HES were selected following a previous screening study [24]: TOPO:Men 1:2, TOPO:DodA 1:1, Men:OleA 2:1, and OctA:DodA 3:1. TOPO acts as an HBA in both HES [25], while Men acts as an HBD with TOPO and as an HBA with oleic acid [26]. In the DodA:OctA system, despite the acidity difference suggesting preferential HBD and HBA roles, respectively, both acids are better regarded as mixed HBA/HBD species [27].

The four HES showed low viscosity (≤50 mPa s), a large density difference with respect to water (≥75 kg m^−3^), low leaching into water (≤0.065 wt%, after a blank test consisting of 1 h of stirring at 25 °C with a HES‐to‐water ratio of 1:1), and an excellent EcoScale score (≥93). These key criteria make them promising systems with low cross‐contamination, high processability, and a favorable environmental profile. Each HES was prepared according to the heating method: the two components were weighed into a screw‐capped sample vial using an analytical balance (OHAUS Explorer, precision = 0.1 mg), mixed at the desired mole fractions, and then heated up to 50 °C under stirring at 800 rpm, using a thermoregulated bath (VFT Fuzzy Logic Thermoregulator, Fisherbrand), until a homogeneous liquid was formed and then stirred in the liquid state for almost 30 min. The eutectic composition as described in the literature from the solid–liquid equilibrium diagrams was used for all HES [25, 27, 28, 29].

### 2.3 Preparation of HEG

The corresponding HEG were prepared in screw‐capped glass vials by weighing the proper amounts of the DBS and 0.5 g of HES. A DBS content of either 2 or 5 wt% was used to ensure the formation of homogeneous matrices with favorable mechanical properties (storage and loss moduli above 10^4^ Pa) according to a previous study [14]. The sample vial was heated in an oil bath between 120 and 135 °C while stirring at 900 rpm, until the solution became clear and transparent. The thermal stability of the HES components is consistent with their use under the temperature conditions employed for DBS dissolution, being the degradation temperatures reported by TGA above the maximum preparation temperature for all HES components used in this study: Men, 169–185 °C [30, 31]; TOPO, 203 °C [32]; OctA, 150 °C [33]; DodA, 292 °C [30]; and OleA, 260–287 °C [34]. The liquid was then poured into a metal cylinder (12‐mm diameter, 10‐mm height) and cooled at room temperature until complete gelation. Gel formation was assessed by the rheological behavior (storage modulus G′ > loss modulus G″). The prepared systems are summarized in Table 1, and representative pictures are given in the Supporting Information (Figure S1).

**TABLE 1:** HEG used in this work.

HES	DBS, wt%	Abbreviations
Trioctylphosphineoxide:DL‐menthol 1:2	2	TOPO:Men‐DBS2
Trioctylphosphineoxide:DL‐menthol 1:2	5	TOPO:Men‐DBS5
Trioctylphosphineoxide:dodecanoic acid 1:1	2	TOPO:DodA‐DBS2
Trioctylphosphineoxide:dodecanoic acid 1:1	5	TOPO:DodA‐DBS5
DL‐Menthol:oleic acid 2:1	2	Men:OleA‐DBS2
DL‐Menthol:oleic acid 2:1	5	Men:OleA ‐DBS5
Octanoic acid:dodecanoic acid 3:1	2	OctA:DodA‐DBS2
Octanoic acid:dodecanoic acid 3:1	5	OctA:DodA‐DBS5

### 2.4 Rheology

The rheological characterization was carried out on an Anton Paar MCR502 rheometer. Tests were performed in oscillation, using a plate–plate configuration with commercially available serrated 25‐mm plates to avoid slippage during measurements. Temperature was controlled with a Peltier plate and hood system (H‐PTD–P‐PTD 200). Tests were performed on disks produced by pouring the hot precursor solution into a rubber O‐ring over a non‐adhesive paper, allowing complete gelation and die‐cutting the formed gel in the appropriate dimension. After positioning the sample, a force of 1 N was applied, leading to a measuring gap ranging between 1.0 and 1.8 mm, depending on the initial sample thickness; then, the gel was left to creep to a stable gap and reach a constant temperature for 5 min before testing. Frequency sweep tests in the angular frequency range of 1–100 rad/s were then performed, with a shear strain amplitude of 0.1% for all HEG except TOPO:Men‐DBS5 and TOPO:DodA‐DBS5 for which a shear strain amplitude of 0.01% was used (within the linear viscoelastic regime, as determined in preliminary strain sweep tests at 10 rad/s, see Figure S2). Measurements were performed at least in triplicate, and results are given as the average (Figures S3 and S4). All tests were carried out at 25 °C and under a constant normal load of 1 N. In all tests, G′ revealed higher than G″, with a very weak dependence of both properties on frequency. This confirmed that the behavior of the eutectogel can be approximated as rubbery elastic. The theory of the entropic elasticity of rubber was therefore applied to estimate the average mesh size or correlation length (ξ, nm). Given the elastic component of the elastic G′ is always at least one order of magnitude higher than G″, it was assumed as a good approximation of the fully relaxed modulus, $G_{0}$, and ξ was calculated from Equation (1) [14, 35]:

(1) $$\xi ={\left(\frac{G^{'}N_{A}}{RT}\right)}^{-1/3}$$

with $N_{A}$ the Avogadro’s constant (6.022 × 10^23^), R the gas constant (8314 JK^−1^ mol^−1^), and T the temperature (here 298 K). Given the weak dependence of G′ on frequency, its average value over the considered frequency range was used in Equation (1).

### 2.5 HRMAS NMR Measurements

The TOPO:Men eutectogels gels were studied by high‐resolution magic‐angle spinning (HRMAS) NMR spectroscopy [36] under different conditions: freshly prepared, after contact with water, and after BPA extraction. HEG samples were transferred in a 4‐mm ZrO_2_ rotor (12.5 μL) and gently packed to ensure homogeneous filling and minimize trapped air bubbles. The upper Kel‐F insert was then positioned to close the sample cavity, the MAS thread pin screwed into place to secure the sample, and a Kel‐F drive cap fitted to the rotor. HRMAS NMR measurements were performed at 298 K at a spin rate of 4 kHz using a Bruker NEO 500 console (11.74 T) (^1^H resonance frequency of 500.13 MHz) equipped with a dual ^1^H/^13^C HRMAS probehead. The use of a relatively low spin rate, a controlled temperature unit, and rotors with reduced volume ensured gel stability under spinning. Spinning sidebands were readily distinguishable from the signals of interest and did not affect spectral assignment or quantitative analysis.

1D ^1^H HRMAS spectra were collected with 64 scans using 16384 points, over a spectral width of 20 ppm. Pulsed‐field‐gradient (PFG) ^1^H HRMAS NMR was applied to determine the self‐diffusion coefficients ($D_{i}$) of the individual species of HEG systems. The self‐diffusion coefficients were determined at 298 K using the bipolar pulse longitudinal eddy current delay (BPP‐LED) pulse sequence. All experiments were carried out with a total of 8 transients per increment using 16384 points in the F_2_ dimension, over a spectral width of 12 ppm. The relaxation delay was set to at least five times T_1_, and 8 dummy scans were programmed prior to acquisition. A pulsed gradient unit producing magnetic field pulse gradients in the *z*‐direction of 53 G cm^−1^ was used. The pulse gradients were incremented from 2% to 95% of the maximum gradient strength in a linear ramp with 32 steps. For each experiment, the duration of the magnetic field pulse gradients (δ) and the diffusion times (Δ) were optimized to obtain 95% signal attenuation for the slowest diffusion species at the last‐step experiment. *δ* value was set at 6.0 ms, while Δ values were 0.45–0.50 s long. The raw spectra acquired were subjected to manual phasing and automatic baseline correction. Data were processed using an exponential filter in the F_2_ dimension (LB = 0.3 Hz). Integrals were employed in the Bruker T_1_/T_2_ module of TopSpin to fit the Stejskal–Tanner relation [37]:

(2) $$\frac{I}{I_{0}}=e^{\left[-D\gamma^{2}g^{2}\delta^{2}\left(\text{Δ}-\frac{\delta }{3}\right)\right]}$$

where I is the signal intensity with the gradient applied, $I_{0}$ is the initial signal intensity, γ is the gyromagnetic ratio of the investigated nucleus, g is the gradient strength applied.

Isolated peaks corresponding to protons of each HES component (Men and TOPO) were integrated, and the corresponding areas were used for the fitting procedure. Specifically, the signals corresponding to protons M1, M7, and M2a of Men, and the signal assigned to protons T3–T7 of TOPO, were integrated (Figure S6). The average value obtained from the three Men signals was used as the diffusion coefficient of Men. The exchangeable hydroxyl signal M10 was treated separately. The remaining peaks were excluded because they resulted from overlapping signals corresponding to both Men and TOPO protons. The complete spectral assignment is provided in the Supporting Information (Figure S6). All signals were fitted with a mono‐exponential function.

Diffusion data for HEG were acquired in at least duplicate using different aliquots of the same HEG to fill at least two rotors. Results are reported as the mean, with error bars representing the standard deviation of individual measurements.

^1^H spectra and diffusion coefficients were compared with the neat HES TOPO:Men. The HES sample was transferred to 5‐mm NMR tubes, equipped with a coaxial insert containing deuterated dimethylsulfoxide (DMSO‐d_6_) and tetramethylsilane (TMS), as the lock signal and chemical shift reference, respectively. High‐resolution liquid‐state NMR measurements were performed at 298 K without sample spinning using a direct observe BBFO (broadband including fluorine) iProbe and a variable‐temperature unit.

### 2.6 Hydrophobicity

The hydrophobic character of the prepared HEG containing 5 wt% DBS was assessed by water uptake experiments. Cylindrical HEG samples (approximately 0.4 g), prepared as described in Section “Preparation of Hydrophobic Eutectogels,” were fully immersed in deionized water in 9‐cm‐diameter Petri dishes and stored at room temperature for 3 days. At 24 h intervals, each sample was removed from the water, gently blotted with a filter paper to remove excess surface water, and weighed. Water uptake was determined from the variation in the sample mass over time. All experiments were performed in duplicate.

### 2.7 Solid–Liquid Extraction (SLE) of BPA

An aqueous solution of BPA with a concentration of 100 ppm was prepared gravimetrically as a controlled model system. The BPA‐contaminated water and cylindrical HEG samples (approximately 0.5 g) were put into contact in a test tube at a HEG:water ratio of 1:4 w/w and mixed for either 1 or 16 h on a hotplate magnetic stirrer Velp Scientifica Arex 5 at 900 rpm or on a LBX Orb‐Pro orbital shaker at 280 rpm, in a condition of 298 K and atmospheric pressure. The 1 h extraction time was selected to compare the HEG performance under a relatively short contact period, while the 16 h experiment was introduced for the most promising systems specifically to determine whether slower mass transfer could be overcome by longer extraction times.

The aqueous phases were separated, and the quantification of BPA as well as the HEG precursor leaching was carried out by ^1^H quantitative NMR (qNMR) spectroscopy. Each experiment was performed in triplicate. qNMR spectra were acquired on a Bruker Avance III 400 MHz spectrometer, operating at a magnetic field strength of 9.4 T, equipped with a PABBO probe‐head. Samples were prepared by adding 500 µL of the aqueous phase after the SLE experiment into a standard 5 mm NMR tube, already filled with 50 µL of a TSP (trimethylsilylpropanoic acid) aqueous solution at a known concentration (11.6 mM), which served as an internal reference. Spectra were recorded at 300 K with 64 k data points, 128 scans, and a 4 s relaxation delay. Water presaturation was achieved using the standard NOESY presaturation scheme (Bruker pulse program noesypr1d). Spectra were processed using an exponential line broadening (LB) of 2 Hz, manual phase correction, and automatic baseline. Isolated signals corresponding to different protons of each species (BPA, HES precursors and DBS) were integrated and the resulting areas were used for quantification. The selected signals were chosen to minimize overlap with signals from the other components and with the residual water signal. Representative ^1^H NMR spectra with selected proton signals explicitly highlighted are reported in the Supporting Information (Figure S5).

The concentrations were calculated as follows:

(3) $$C_{\text{sample}}=\frac{I_{\text{sample}}.N_{\mathrm{ref}}}{N_{\text{sample}}.I_{\mathrm{ref}}}C_{\mathrm{ref}}$$

where $C_{\text{sample}}$ and $C_{\mathrm{ref}}$ are the unknown concentration of the analyte (BPA or HEG species) and the known concentration of TSP (1.05 mM) in the NMR sample, $I_{\text{sample}}$ and $I_{\mathrm{ref}}$ are the integrated areas of the proton signals for the analyte and TSP, and $N_{\text{sample}}$ and $N_{\mathrm{ref}}$ are the number of protons associated to each signal for the analyte and TSP. The LOD and LOQ were calculated at signal‐to‐noise (S/N) ratio of 3 and 9, respectively.

The extraction efficiency (EE) for BPA removal was calculated from the residual concentration of the contaminant in the water phase according to Equation (4):

(4) $$\mathrm{EE}\;\left[\%\right]=\frac{C_{\mathrm{BPA}}^{i}-C_{\mathrm{BPA}}^{f}}{C_{\mathrm{BPA}}^{i}}\cdot 100$$

where $C_{\mathrm{BPA}}^{i}$ and $C_{\mathrm{BPA}}^{f}$ are the initial and final concentrations of BPA in the water phase, respectively.

The leaching of the HEG component(s) in the water phase (Leach(i), i being the HEG component) was calculated from the residual concentration of the given species in the water phase according to Equation (5):

(5) $$\text{Leach}(i)\;\left[\%\right]=\frac{{m(i)}_{\text{water}}^{f}}{{m(i)}_{\mathrm{HEG}}^{i}}\cdot 100$$

where ${m(i)}_{\text{water}}^{f}$ is the final mass (in mg) of each HES component i in the water phase after extraction and ${m(i)}_{\mathrm{HEG}}^{i}$ is the mass (in mg) of the same component i in the HEG before the extraction.

In TOPO:Men and Men:OleA, selected proton signals of Men, TOPO, and OleA appeared in separate spectral regions and were therefore individually integrated. This allowed the quantification of Men leaching, whereas TOPO and OleA concentrations were below the quantification limit. Therefore, Leach(Men) was reported as the maximum leaching value for these systems. In OctA:DodA and TOPO:DodA, the signals of the two components overlapped in the corresponding ^1^H NMR spectra, preventing their individual quantification. The leaching value was therefore calculated by integrating the overlapping signals arising from both precursors and reported as an average value Leach(HES). In all systems, DBS leaching could be individually quantified.

A schematic workflow showing the experimental procedure (HES preparation, DBS incorporation, HEG formation, solid–liquid extraction, phase separation, and NMR analysis) is depicted in Scheme S1.

## 3 Results and Discussion

### 3.1 Preliminary Characterization of the Novel Supramolecular HEG

A set of four HES were prepared mixing a terpene (Men), a phosphine oxide (TOPO), and three long‐chain carboxylic acids (OctA, DodA, and OleA) in the following combinations (Table 1): TOPO:Men (1:2), TOPO:DodA (1:1), Men:OleA (2:1), and OctA:DodA (3:1). According to a previous screening [24], the four systems are promising candidates for extraction applications in the water phase, thanks to their low viscosity (7–50 mPa s), high density difference with respect to water (75–95 kg m^−3^), low leaching into the water phase (0.015–0.065 wt%), chemical stability over 1 month (no change in the ^1^H NMR spectra or viscosity), and high EcoScale score (93–95). Importantly, these systems provide a chemically diverse platform, allowing the influence of different intermolecular interactions within the HES phase on the final HEG performance to be investigated.

For each HES, two HEG were prepared at DBS concentration of 2 and 5 wt%. The hydrophobic nature was confirmed by immersing the eutectogels with 5 wt% DBS in water and observing no mass increase in any HEG over 3 days of immersion (Figure 2a). Rather, weight losses up to 5 wt% of the initial HEG mass were observed for OctA:DodA and Men:OleA HEG, while TOPO:Men‐ and TOPO:DodA‐based HEG stood out as the most stable systems. These differences are attributed primarily to differential component leaching rather than water absorption and well align with the solubility‐driven leaching data previously collected for the neat HES, in which virtually no leaching was observed for TOPO‐containing HES, whereas OctA:DodA and Men:OleA displayed low, albeit non‐negligible, leaching [24]. Notably, the weight losses measured here at 24 h for all four systems are between 2.3% for OctA:DodA‐DBS5 and 0.4% for TOPO:Men‐DBS5, thus showing higher or comparable stability than that of recently reported polymeric eutectogels [38]. This high stability in water supports the use of supramolecular gelation as a straightforward and efficient strategy for eutectogel fabrication.

**FIGURE 2:**
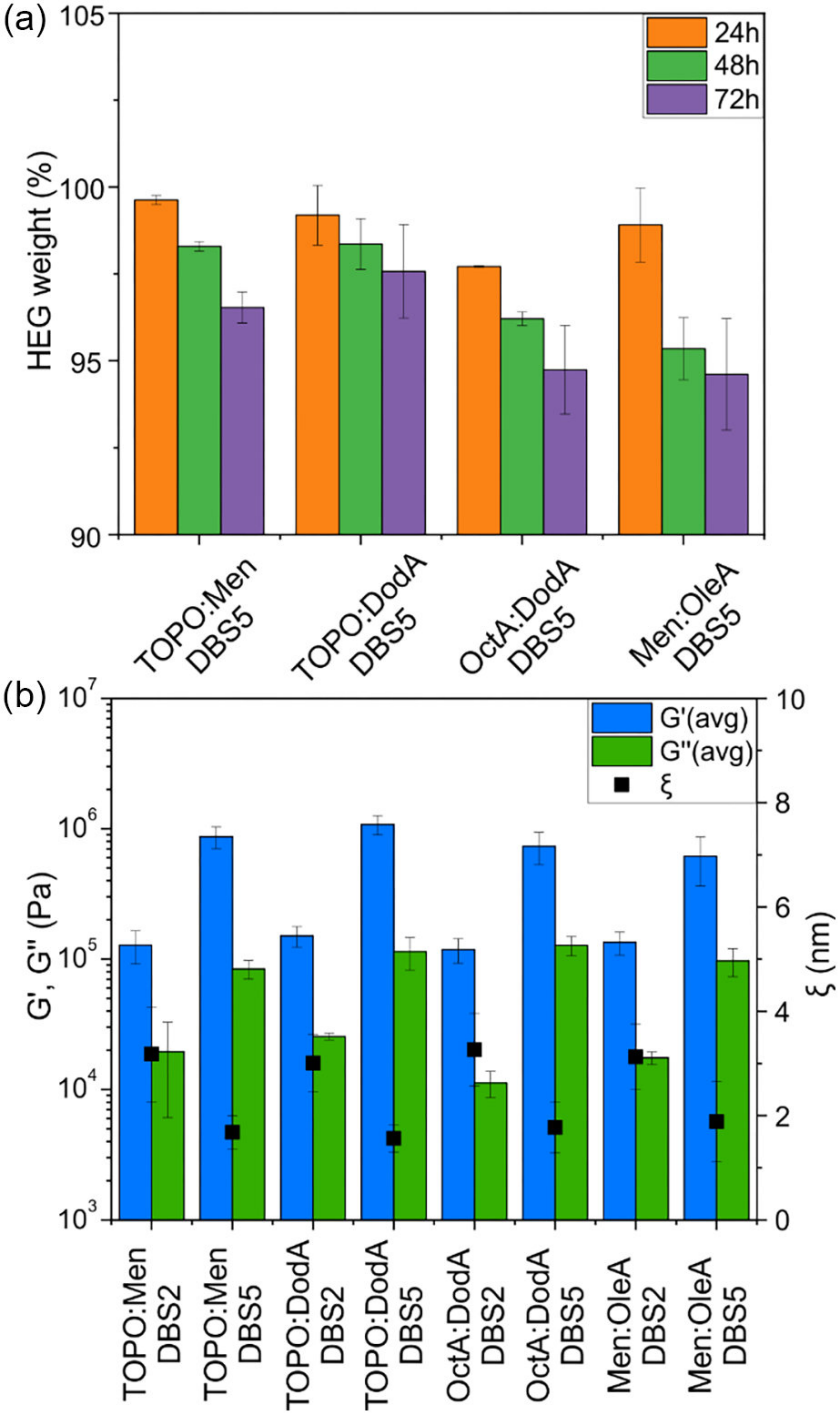
(a) Weight loss data obtained for the four HEG at 5 wt% DBS content in contact with water for 3 days at 24‐h intervals. (b) Average storage modulus (G′, blue bars) and loss modulus (G″, green bars) measured for the eight HEG at 25 °C during the angular frequency sweep, and calculated mesh size from Equation (1) of the Experimental Section (black squares). Error bars represent standard deviation across replicates.

Rheological measurements were performed on the eight HEG molded into disk‐shaped samples. The frequency sweep experiments reported in Figure S3 confirm that all HEG are real gels with the storage modulus G′ about one order of magnitude larger than the loss modulus G″. The measured G′ values were only slightly dependent on the angular frequency, further confirming the formation of a gel. Figure 2b shows the values of G′ and G″ measured at 25 °C during the angular frequency sweep. As G′ and G″ were only slightly dependent on the angular frequency, the average values are used for comparison. It can be observed that the rheological behavior is substantially controlled by the DBS content, with almost no differences stemming from the four HES. Comparing the HEG with 5 wt% DBS, TOPO‐based systems show slightly higher average storage moduli (868 and 1080 kPa for TOPO:Men and TOPO:DodA, respectively) than the alcohol:acid and acid:acid systems (614 and 734 kPa for Men:OleA and OctA:DodA, respectively). Overall, storage moduli of 110–150 kPa for the 2 wt% DBS and 600–1000 kPa for the 5 wt% DBS have been measured, which are comparable with previous data on similar DBS‐based eutectogels assembled in a number of HES [7, 14]. The present eutectogels are slightly stiffer than DBS‐gels formed in ionic liquids (storage moduli of 7–85 kPa at DBS 3%) [12] and more than 2 orders of magnitude stiffer than recently reported polymeric eutectogels (storage moduli up to 800 Pa) [38]. The average network mesh size (ξ) was calculated according to Equation (1) (Table S2), assuming to a first approximation that the gels behave as ideal crosslinked systems above their glass transition. Given the weak dependence of G′ on frequency, its average value over the considered frequency range was used in Equation (1). The values obtained are close to values previously obtained for similar HEG containing the same DBS amount [7, 14], with values of 3.1–3.2 nm for 2% DBS and 1.7–1.9 for DBS 5%. Overall, the rheological data indicate that a higher DBS concentration results in a higher storage modulus and a smaller estimated mesh size. This, in turn, translates into a denser supramolecular network and an increased molecular confinement.

### 3.2 SLE of BPA

Among the commonly used methods for BPA removal from water bodies, including nanofiltration, reverse osmosis, advanced oxidation processes, and membrane bioreactors, sorption has risen to some prominence due to its simple operating design, relatively low energy consumption, cost‐effectiveness, low production of harmful byproducts, and, in many cases, high removal efficiency [21, 39]. While a number of studies investigated the potential of HES‐containing polymeric gels and membranes for water remediation [38, 40, 41, 42, 43], only a few explored supramolecular noncovalently linked eutectogels [10, 44, 45]. Supramolecular HEG can represent a novel cost‐effective and sustainable alternative to the conventional and less common sorbents currently available (such as clays, nanomaterials, graphene, activated carbons, imprinted polymers, agricultural wastes) [21] for BPA removal that combine the innate dissolution capabilities of HES with the greater ease of recovery of gels. Their supramolecular gel structure potentially provides both mechanical stability, facilitating handling, separation and recovery after use, and high surface areas, increasing contact with the pollutant and effective sorption. In parallel, the hydrophobic nature and already proved affinity of the selected HES toward BPA allow the innovative materials to repel water while selectively interacting with BPA. Moreover, the variety of starting materials and possible combinations to prepare the DBS‐based HEG offers a versatile tool for developing tailor‐made materials for specific target pollutants in water treatment processes.

In the present work, all eight HEG formulations were tested in SLE experiments of BPA 100 ppm, and the residual contaminant as well as the HES or DBS leaching in the water phase was monitored via qNMR measurements using TSP as an internal reference (see the Experimental Section). Isolated signals corresponding to protons of BPA, DBS, and HES components were chosen for integration and quantification, minimizing overlap with signals from the other components and with the residual water. Representative ^1^H spectra are reported in Figure S5. Given the fast equilibrium achievable in liquid–liquid extraction with neat HES [24], the effect of the agitation step was first evaluated, with either orbital shaker or magnetic stirring used, to enhance the mass transfer during the extraction time of 1 h. Tables S3–S6 and Figure 3 show that 1 h extraction time is not sufficient to reach near‐complete BPA removal for all systems and agitation conditions, indicating that equilibrium is reached more slowly than the LLE, with both shaker and stirrer.

**FIGURE 3:**
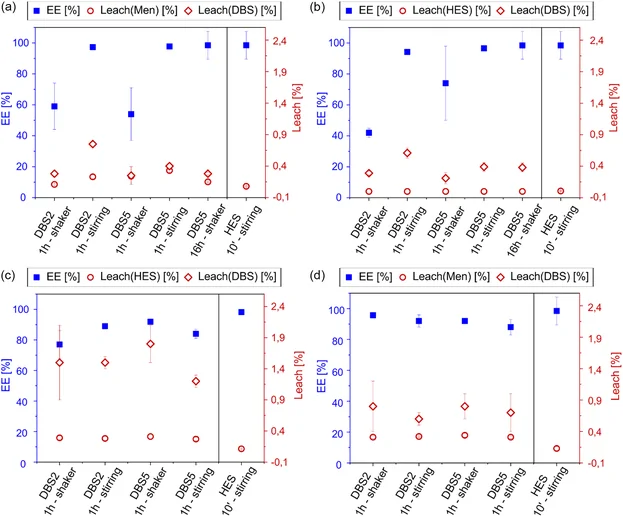
BPA extraction efficiency (EE, %) and HEG species leaching (Leach(i), with i the HEG component, %), using (a) TOPO:Men‐DBS, (b) TOPO:DodA‐DBS, (c) OctA:DodA‐DBS, and (d) Men:OleA‐DBS, under various experimental conditions. Comparison with the liquid HES from ref. [24] (HES, 10 min, stirring) is also shown. Error bars represent standard deviation across replicates. Where not visible, error bars are smaller than the marker size.

Both TOPO‐containing systems (Figure 3a,b) exhibit a clear increase in extraction when going from the orbital shaker (EE% between 42% and 74%) to magnetic stirring (from 94.2% to 97.7%), with EE% in DBS5 systems at 1 h stirring very close to the liquid–liquid extraction performed in 10 min under magnetic stirring (approximately 97% in SLE versus > 98.5% in LLE). This is reasonably explained considering that magnetic agitation ensures a more efficient mass transfer than the orbital shaker during the same contact time. Contrarily, the extraction performance of OctA:DodA and Men:OleA (Figure 3c,d) is rather independent of the DBS content and agitation mechanism, with EE% consistently high (above 77% for OctA:DodA and 92% for Men:OleA) but below the performance of the neat HES.

In addition to the EE%, another crucial parameter is the cross‐contamination due to the leaching to the water phase of both HES components and DBS. A markedly different behavior was again observed for the TOPO‐based HEG when compared to OctA:DodA and Men:OleA. In the former sorbents (Figure 3a,b), there is a clear and reasonable increase of HEG component leaching when going from an orbital shaker to magnetic stirring. For instance, in TOPO:Men‐DBS, Men leaching increases from 0.11 to 0.23 wt% for 2 wt% DBS and from 0.24 to 0.33 wt% for 5 wt% DBS. Similarly, DBS leaching increases from 0.28 to 0.75 wt% in TOPO:Men‐DBS2 and from 0.29 to 0.61 wt% in TOPO:DodA‐DBS2. Moreover, a reduction of DBS leaching was observed when passing from a DBS content of 2–5 wt% (from 0.75 to 0.40 wt% in TOPO:Men under stirring and from 0.61 to 0.39 wt% from TOPO:DodA under stirring). These trends can be reasonably explained considering that (i) agitation via magnetic stirring promotes the migration of menthol and DBS to the water phase more remarkably than a gentle shaking movement without magnetic bars and (ii) stiffer gels with higher DBS content create a more connected DBS network, thus reducing the gelator leaching to the water phase. The behavior of OctA:DodA and Men:OleA HEG is less clear (Figure 3c,d), with DBS and HES leaching substantially independent of both the DBS content and the agitation step. Moreover, the DBS leaching was consistently higher across all SLE conditions for the latter two HEG (in the range of 0.6–1.8 wt%), which prompted us to disregard these two systems. Although the present experiments cannot, by themselves, unambiguously distinguish molecular dissolution of individual HES components from release associated with local structural rearrangement or structural degradation of the supramolecular network, the absence of major changes in rheological properties and the preservation of characteristic HRMAS NMR signals after water contact/BPA extraction (vide infra) indicate that the observed leaching is not accompanied by detectable structural degradation of the gel network.

The different performance of the TOPO‐based HEG with respect to the acid:acid and Men:acid HEG can be linked to the different molecular organization of the HES and its response to the supramolecular DBS network. The lone HBA nature and highly directional H‐bond‐accepting ability of the phosphine oxide group of TOPO, together with the dispersive forces existing between the octyl chains, generate strong and more localized interactions [25, 46]. In the alcohol–acid and acid–acid HES, both components may act as HBA, creating a more distributed and likely dynamic H‐bond network. We hypothesize that, when organizing in its supramolecular network in TOPO‐based HEG, DBS fibrils would confine TOPO‐centered microdomains, where the local organization reflects the original one. This interpretation could rationalize both the reduced leaching and the need for a stronger agitation mechanism to force the interaction rearrangement and the following BPA extraction. In other words, a more strongly organized and mechanically robust network would suppress component release, while simultaneously imposing greater resistance to molecular rearrangement and mass transfer. Contrarily, in Men:OleA and OctA:DodA HEG, the DBS fibrils span through a distributed and more dynamic H‐bond network, where the rearrangement of all interactions occurs more easily. This enables on the one side the establishment of novel interactions with BPA even with a gentle mass transfer ensured by orbital shaking, and on the other a more significant leaching of the HES component to the water phase. The proposed molecular interpretation is also consistent with the stability and rheological data described above, with TOPO‐driven strong and localized interactions responsible for the formation of more stable gels with respect to the alcohol–acid and acid–acid HES.

In order to enhance the extraction, while limiting leaching, a further test was carried out with the orbital shaker at longer extraction times (16 h) for the most promising TOPO‐based HEG at 5% DBS. Tables S3 and S4 and Figure 3a,b show that a complete removal (above the NMR detection limit) was reached for both TOPO:Men‐DBS5 and TOPO:DodA‐DBS5, with Men and DBS leaching comparable to a shorter extraction time (1 h). Among the two adsorbents, TOPO:DodA‐DBS5 stands out for the absence of HES leaching.

The excellent sorption ability found here for the TOPO‐based HEG is comparable to results obtained with polymeric PEGDA‐HA (poly(ethylene glycol) diacrylate‐ethyl hexylacrylate) eutectogels containing octanoic acid menthol HES (BPA extraction of roughly 97.5%) [38], and DBS‐gels assembled in imidazolium, pyrrolidinium, and piperidinium ionic liquids [12], and even better than porous PVDF membranes loaded with dodecanoic acid:decanoic acid 1:2 (62.7% BPA extraction, after 20 min of contact time) [40]. Even considering the differences in the extraction setup, these findings support further investigation of supramolecular HEG as simple and efficient BPA sorbents. Moreover, the cross‐contamination due to HES leaching is limited compared to structurally related DBS‐gels obtained in ionic liquids (0.3–1.0 wt%) [12], and to the best of our knowledge, this is the first work putting the accent on the DBS leaching, thus suggesting accurate monitoring as a crucial parameter in the field.

Beyond extraction performance, the sustainability profile of the proposed approach was also considered to align to Green Analytical Chemistry (GAC) and the principles of Green Sample Preparation (GSP) [47]. Despite the lab scale of the process, both systems are ranked as excellent in the Analytical EcoScale metrics (score > 75) [48], with scores equal to 86 and 85 for TOPO:Men‐DBS5 and TOPO:DodA‐DBS5, respectively, the only difference being related to the reagent hazard (see the Supporting Information).

### 3.3 Molecular and Macroscopic Insights Into the HEG Response Under Operation

To evaluate the effect of water contact and BPA uptake on the proposed HEG, the molecular diffusion and rheological behavior were monitored for the representative TOPO:Men HEG.

As observed for other hydrophobic DBS‐based eutectogels [13, 14], ^1^H liquid‐state and high‐resolution magic angle spinning (HRMAS) NMR spectra of neat TOPO:Men and HEG with DBS at 2 and 5 wt% are very similar (Figure S6), indicating that the HES in the HEG retains its pristine liquid‐like nature. Rather than immobilizing the HES, the DBS network confines a liquid phase, demonstrating that supramolecular gelation and solvent mobility coexist within the same material. In addition, no evident changes are observed in the 1D and ^1^H spectra after contact with water or BPA uptake (Figure 4). The translational motion was investigated by measuring the apparent self‐diffusion coefficients for the individual components by PFG‐NMR (Table S9 and Figure 5a). In all samples, Men diffuses faster than TOPO. A peculiar mobility enhancement is observed in the neat HEG TOPO:Men‐DBS2 with respect to the neat liquid HES ($D_{\mathrm{Men}}$ increases from 2.4 × 10^−11^ m^2^ s^−1^ to 3.6 × 10^−11^ m^2^ s^−1^, while $D_{\text{TOPO}}$ from 1.3 × 10^−11^ m^2^ s^−1^ to 2.2 × 10^−11^ m^2^ s^−1^). This counterintuitive DBS‐induced increase in diffusion coefficients has been already observed in both hydrophobic and hydrophilic eutectogels [13, 14, 49, 50] and can be interpreted as the result of the perturbation of the local inter‐HES interactions by the sample‐spanning network established by the supramolecular gelator, resulting in an overall increased mobility of the HES species in the system with respect to the neat HES. As observed for thymol:camphor HEG [14], when the DBS content increases from 2 to 5 wt% the constraints created by the DBS to the free diffusion of the species start reducing the diffusion coefficients, in agreement with the estimated reduction in the mesh size ($D_{\mathrm{Men}}$ decreases from 3.6 × 10^−11^ m^2^ s^−1^ to 3.1 × 10^−11^ m^2^ s^−1^, while $D_{\text{TOPO}}$ from 2.2 × 10^−11^ m^2^ s^−1^ to 1.9 × 10^−11^ m^2^ s^−1^).

**FIGURE 4:**
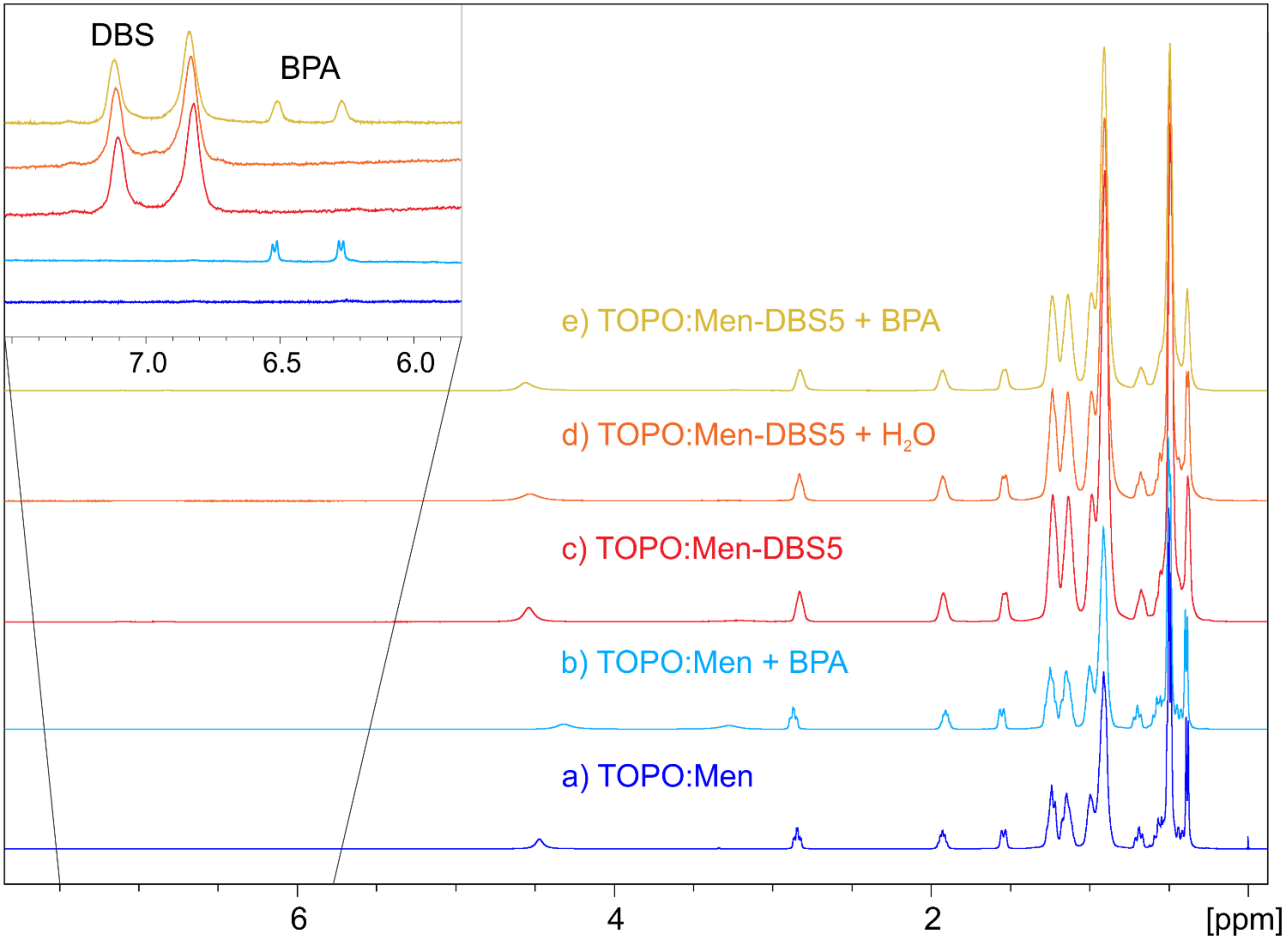
1D ^1^H liquid‐state NMR spectra at 298 K of HES TOPO:Men 1:2 neat (a, blue) and after BPA extraction under stirring for 10 min (b, light blue), and 1D ^1^H HRMAS NMR spectra of HEG TOPO:Men‐DBS5 at different conditions: freshly prepared (c, red), after contact with water under stirring for 1 h (d, orange), and after extraction of BPA under stirring for 1 h (e, yellow). An enlargement of the aromatic region is given, showing signals corresponding to DBS and BPA. The complete spectral assignment is provided in Figure S5.

**FIGURE 5:**
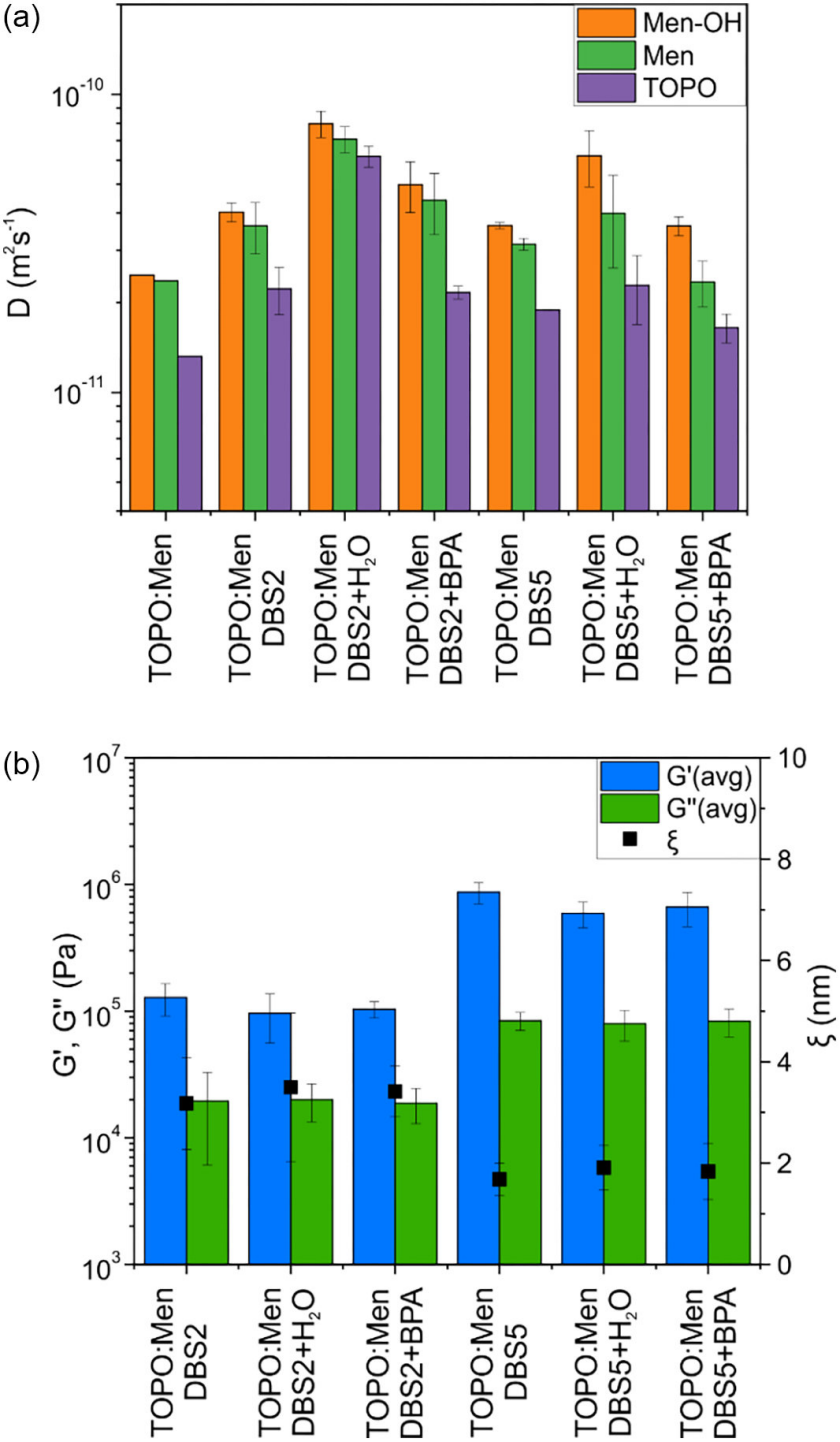
(a) Self‐diffusion coefficients of Men‐OH (orange bars), Men (green bars), and TOPO (purple bars) measured for TOPO:Men HES and HEG at 25 °C under different conditions: freshly prepared (TOPO:Men, TOPO:Men‐DBS2, and TOPO:Men‐DBS5), after water contact (TOPO:Men‐DBS2+H_2_O and TOPO:Men‐DBS5+H_2_O), and after BPA extraction (TOPO:Men‐DBS2+BPA and TOPO:Men‐DBS5+BPA). (b) Storage modulus (G′, blue bars), loss modulus (G″, green bars) measured at 25 °C, and corresponding mesh size (black squares), for the same HES and HEG systems. Error bars represent standard deviation across replicates.

Looking at the effect of the additional water or BPA molecules on the diffusivity of the HES species, it can be observed that, upon contact with water, the diffusion coefficients of both Men and TOPO increase, with a more prominent effect on the exchangeable OH proton of Men. This might indicate that proton exchange between the Men‐OH protons and water molecules slightly weakens the HES intermolecular network, with an overall increase in the diffusivity of all species. This effect is less pronounced in TOPO:Men‐DBS5 HEG, where the enhanced exchange rate of the Men‐OH protons mainly results in an increase in the diffusion coefficient of the exchangeable protons while only slightly affecting the Men diffusivity and even less the TOPO diffusivity. This is consistent with the dense DBS network providing strong confinement. After BPA extraction, all diffusion coefficients return to values close to those measured in the neat systems, an effect which is more evident with DBS at 5 wt%. Overall, this indicates that the translational motion of the HES components is not compromised upon the uptake of pollutant, enabling the potential reuse of the HEG over multiple extraction cycles.

The absence of detrimental effects resulting from water exposure or contaminant uptake is even more evident when considering the bulk rheological behavior. As shown in Figures 5b and S4, no significant changes in the storage and loss moduli are observed within the experimental uncertainty after HEG use, in agreement with the molecular‐level insights discussed above.

From a molecular viewpoint, BPA contains two phenolic OH groups and an aromatic hydrophobic moiety and can therefore interact with both the HES components and the supramolecular environment. The present NMR data do not allow us to unambiguously identify specific BPA–HES or BPA–DBS interactions. Nevertheless, the return of the diffusion coefficients toward the values of the fresh HEG after BPA extraction, along with the absence of significant changes in the macroscopic rheological properties, suggests that these interactions do not produce a permanent restructuring of the network. Together, these observations indicate that water and BPA can transiently modify molecular mobility without causing a detectable permanent alteration of the macroscopic supramolecular network.

## 4 Conclusions

Supramolecular hydrophobic nonionic eutectogels (HEG) were successfully developed as sorbent materials for the removal of BPA from aqueous environments. By combining hydrophobic nonionic eutectic solvents (HES) with the self‐assembly of the low‐molecular‐weight gelator DBS, liquid extraction phases were converted into mechanically stable, easy‐to‐handle gel systems while preserving the intrinsic mobility of the confined solvent components. The four HES were deliberately selected to provide chemically distinct interaction environments. The TOPO‐containing systems contain a phosphine oxide group acting predominantly as a hydrogen‐bond acceptor, whereas in the alcohol:acid or acid:acid combinations, the intermolecular hydrogen‐bond network is expected to be more distributed and dynamic.

The combined rheological, HRMAS NMR, and extraction studies revealed that BPA uptake does not compromise either the translational mobility of the HES or the mechanical integrity of the supramolecular network, supporting the potential reusability of these materials. The results indicated that the DBS concentration primarily controls the macroscopic stiffness and estimated network mesh size, whereas the chemical nature of the HES determines how the confined liquid phase responds to the DBS network and to the extraction process. Increasing the DBS concentration from 2 to 5 wt% produces a denser network, reflected by higher G′ (600–1000 kPa at 5 wt% DBS versus 110–150 kPa at 2 wt% DBS) and a smaller estimated mesh size (1.6–1.9 nm at 5 wt% DBS versus 3.0–3.3 at 2 wt% DBS). This is in turn associated with stronger confinement and lower molecular mobility of the HES components. This effect is particularly evident for TOPO‐based systems, which also show comparatively low component leaching. While exhibiting high stability and low leaching, TOPO‐based systems required more efficient agitation or longer contact times to reach nearly quantitative BPA removal, whereas the more dynamically organized Men:acid and acid:acid systems show relatively high extraction under gentler conditions but also greater DBS/HES leaching. More importantly, the results demonstrate that the extraction performance of HEG is not solely dictated by the affinity of the parent HES toward BPA. Instead, the supramolecular organization induced by DBS introduces an additional level of control over molecular mobility, mass transfer, pollutant uptake, and leaching, highlighting the crucial role of intermolecular interactions in determining the performance of the final material.

Among the investigated systems, TOPO‐based HEG provided the most favorable balance among BPA extraction efficiency, HES/DBS leaching, and structural stability, achieving >98.5% BPA removal at 5 wt% DBS after 16 h under orbital shaking, while DBS and HES leaching remained comparatively low. The different behavior observed with respect to menthol:acid and acid:acid HES suggests that the localized intermolecular organization promoted by the phosphine oxide functionality is largely preserved within the gel network, ultimately governing both extraction kinetics and resistance to solvent release.

Although the extraction conditions applied here do not reproduce the concentration range or complexity of real wastewater matrices, this work should be regarded as a controlled proof of concept to demonstrate that supramolecular hydrophobic eutectogels constitute more than a simple strategy for immobilizing eutectic solvents. HEG represent a versatile platform in which the molecular organization of the liquid phase can be harnessed to engineer the macroscopic behavior of soft materials. This molecular‐to‐material design concept opens up new opportunities for sustainable water remediation and separation processes. Further efforts will be devoted to monitoring and reducing leaching in any practical application and to assessing performance in more complex wastewater matrices. Moreover, long‐term stability over repeated extraction/regeneration cycles remains to be established, and further toxicological and life‐cycle assessments would be required before practical environmental deployment.

## Funding

This work was supported by the European Union Next Generation EU in the framework of the PRIN 2022 PNRR (P2022J9C3R).

## Conflicts of Interest

The authors declare no conflicts of interest.

## Supporting information

Supplementary Material

## Data Availability

The data that support the findings of this study are available from the corresponding author upon reasonable request.
