# Supplementary material for: DBS‐Based Hydrophobic Nonionic Supramolecular Eutectogels: Bridging Molecular Insights to Water Remediation
Source: ChemistryOpen. 2026 Oct 23;15(11):e70323. doi: 10.1002/open.70323 (PMC13693409; doi:10.1002/open.70323)
Supplement: Supplementary file 1 [file OPEN-15-e70323-s001.pdf]

Supporting Material

DBS-based Hydrophobic Non-Ionic Supramolecular Eutectogels: Bridging Molecular Insights to Water Remediation

Chiara Carotti,[a] Laura Riva,[a] Alberto Mannu,[a]† Arianna Rossetti,[a,b] Gloria Nicastro,[a] Francesco Briatico Vangosa,[a] Andrea Mele,[a] and Maria Enrica Di Pietro*[a]

[a] Department of Chemistry, Materials and Chemical Engineering "G. Natta", Politecnico di Milano, Via E. Bassini 6, 20133, Milan, Italy
E-mail: mariaenrica.dipietro@polimi.it

[b] INSTM—Local Unit c/o Politecnico di Milano, Via E. Bassini 6, Milano, 20133, Italy

† Present address: INSTM and Chemistry for Technologies Laboratory, Department of Mechanical and Industrial Engineering, University of Brescia, Via Branze 38, 25123 Brescia, Italy

Materials

Table S1. List of compounds used in this work.

Compound	Abbreviation	CAS number	Supplier	Purity [a]
DL-Menthol	Men	89-78-1	Sigma-Aldrich	≥ 98 %
Trioctylphosphineoxide	TOPO	78-50-2	TCI chemicals	≥ 95 %
Octanoic Acid, Caprylic Acid	OctA	124-07-2	Sigma-Aldrich	≥ 99 %
Dodecanoic acid, Lauric acid	DodA	143-07-7	Sigma-Aldrich	≥ 99 %
Oleic Acid, cis-9-Octadecenoic acid	OleA	112-80-1	Sigma-Aldrich	≥ 99 %
1,3:2,4-Dibenzylidene-D-sorbitol	DBS	32647-67-9	Accela, Aldrich partner	≥ 95 %
Bisphenol A	BPA	80-05-7	Sigma-Aldrich	≥ 99 %
Deuterated water	D_2O	7789-20-0	Sigma-Aldrich	99.9 atom % D
Dimethyl sulfoxide-d_6	DMSO-d_6	2206-27-1	Sigma-Aldrich	99.9 atom % D
3-(Trimethylsilyl)propionic-2,2,3,3-d_4 acid sodium salt	TSP	24493-21-8	Isotec	98%
Tetramethylsilane	TMS	75-76-3	Sigma-Aldrich	≥ 99.0 %

[a] As stated by the supplier

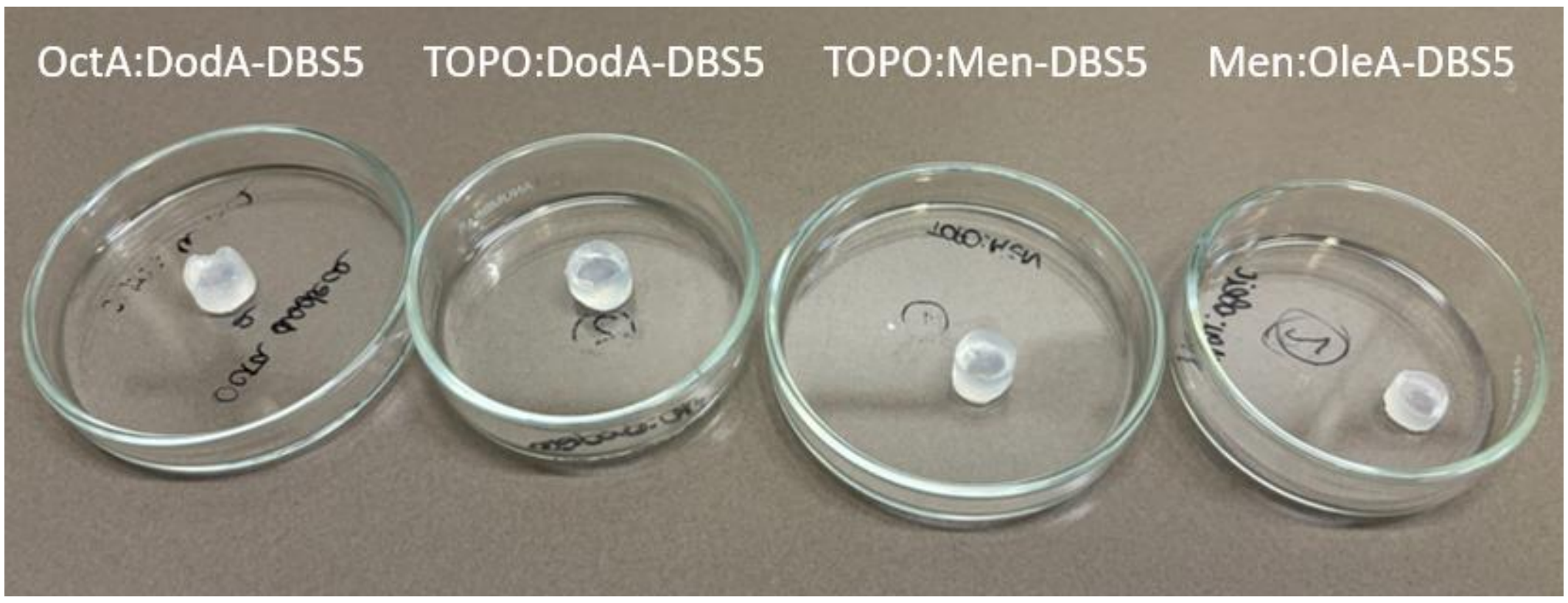

Figure S1. Representative pictures of the HEG containing 5 wt% DBS.

Experimental procedure

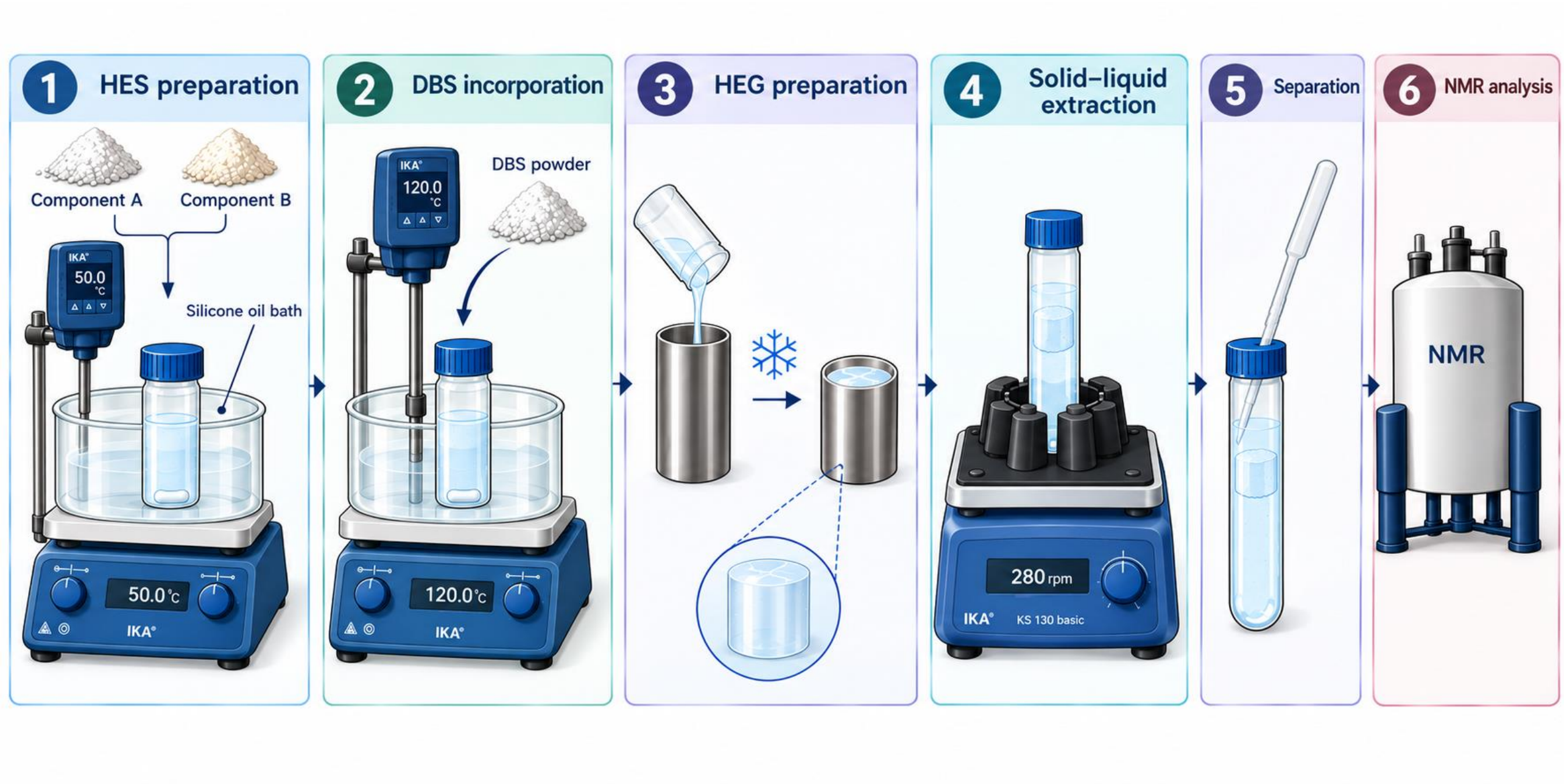

Scheme S1. Schematic representation of the experimental workflow used for HES preparation, DBS incorporation, HEG formation, solid–liquid extraction, phase separation, and NMR analysis. The workflow illustration was created with assistance from OpenAI ChatGPT using its image-generation tool (September 2026).

Rheological data

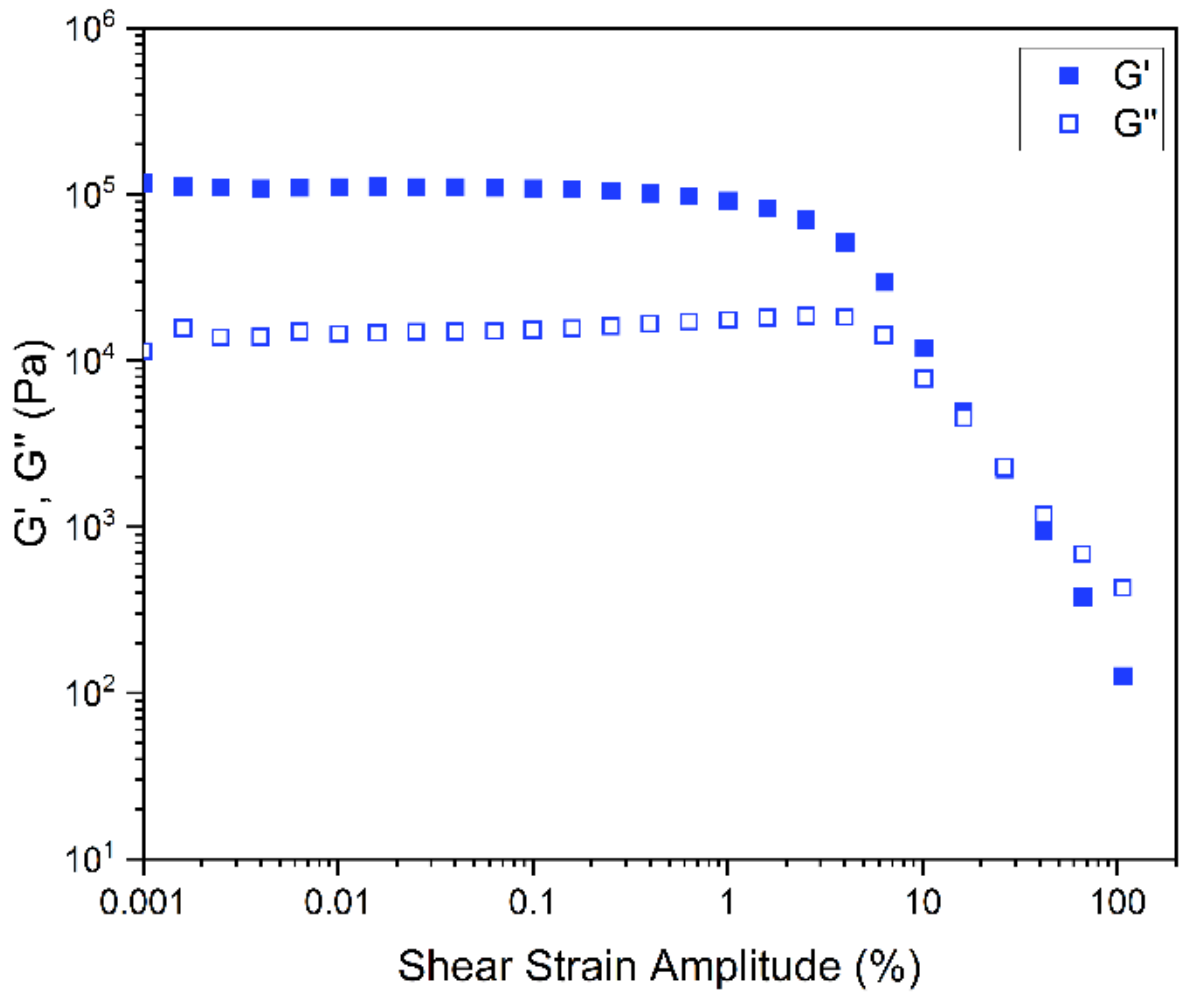

Figure S2. Shear strain dependence of the storage modulus (G', filled markers) and loss modulus (G'', empty markers) measured at 25°C for TOPO:Men-DBS2.

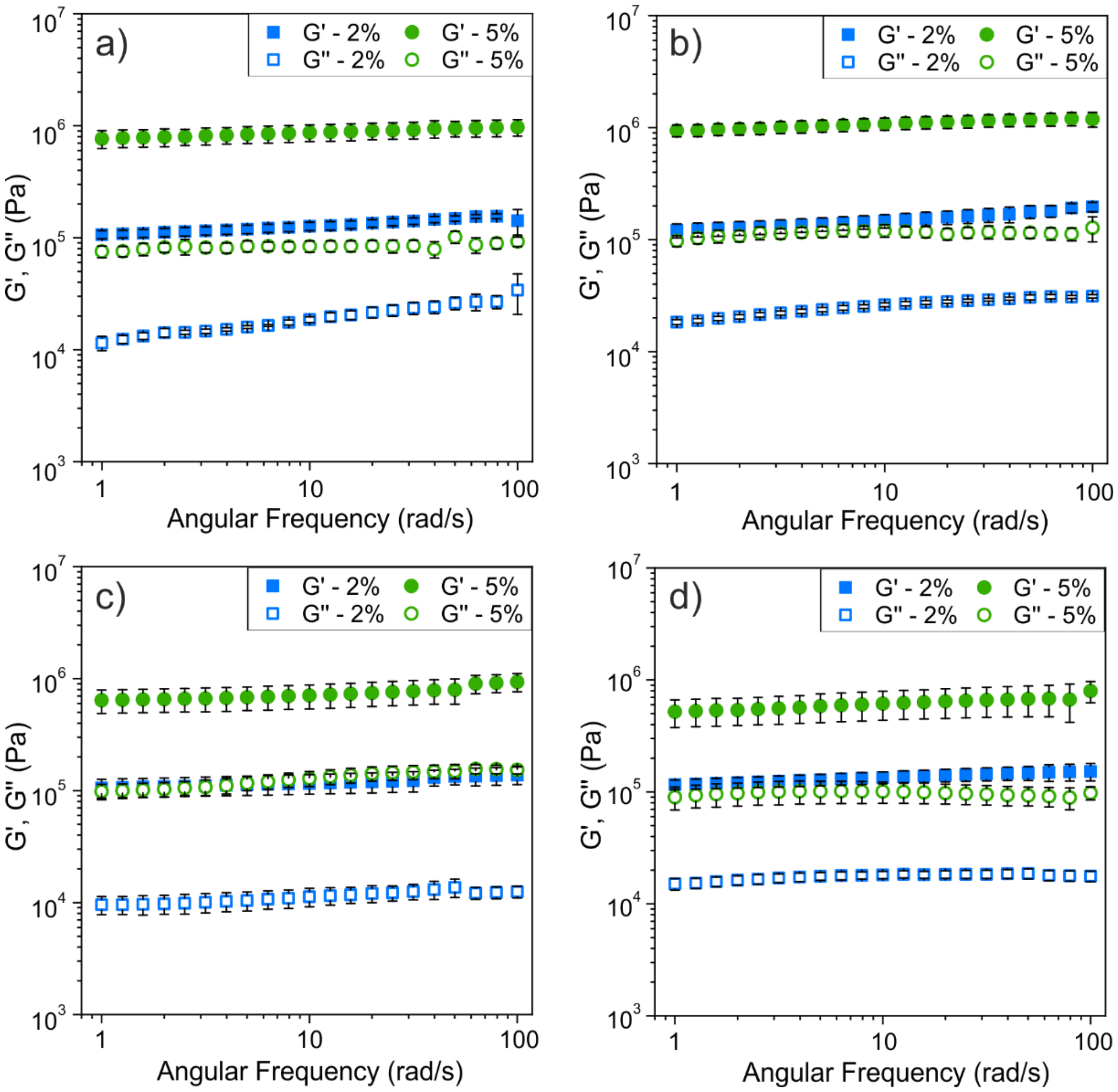

Figure S3. Angular frequency dependence of the storage modulus (G', filled markers) and loss modulus (G'', empty markers) measured at 25°C for TOPO:Men-DBS (a), TOPO:DodA-DBS (b), OctA:DodA (c) and Men:OleA-DBS (d) HEG, with DBS content at 2wt% (blue squares) and 5wt% (green circles). Error bars represent standard deviation across replicates. Where not visible, error bars are smaller than the marker size.

Table S2. Average mesh size ξ (in nm) calculated according to Eq. 1 for all HEG.

HEG	ξ (nm)
TOPO:Men-DBS2	3,18
TOPO:Men-DBS5	1,68
TOPO:DodA-DBS2	3,01
TOPO:DodA-DBS5	1,56
OctA:DodA-DBS2	3,26
OctA:DodA-DBS5	1,78
Men:OleA-DBS2	3,13
Men:OleA-DBS5	1,89
TOPO:Men-DBS2+H_2O	3,50
TOPO:Men-DBS2+BPA	3,41
TOPO:Men-DBS5+H_2O	1,91
TOPO:Men-DBS5+BPA	1,84

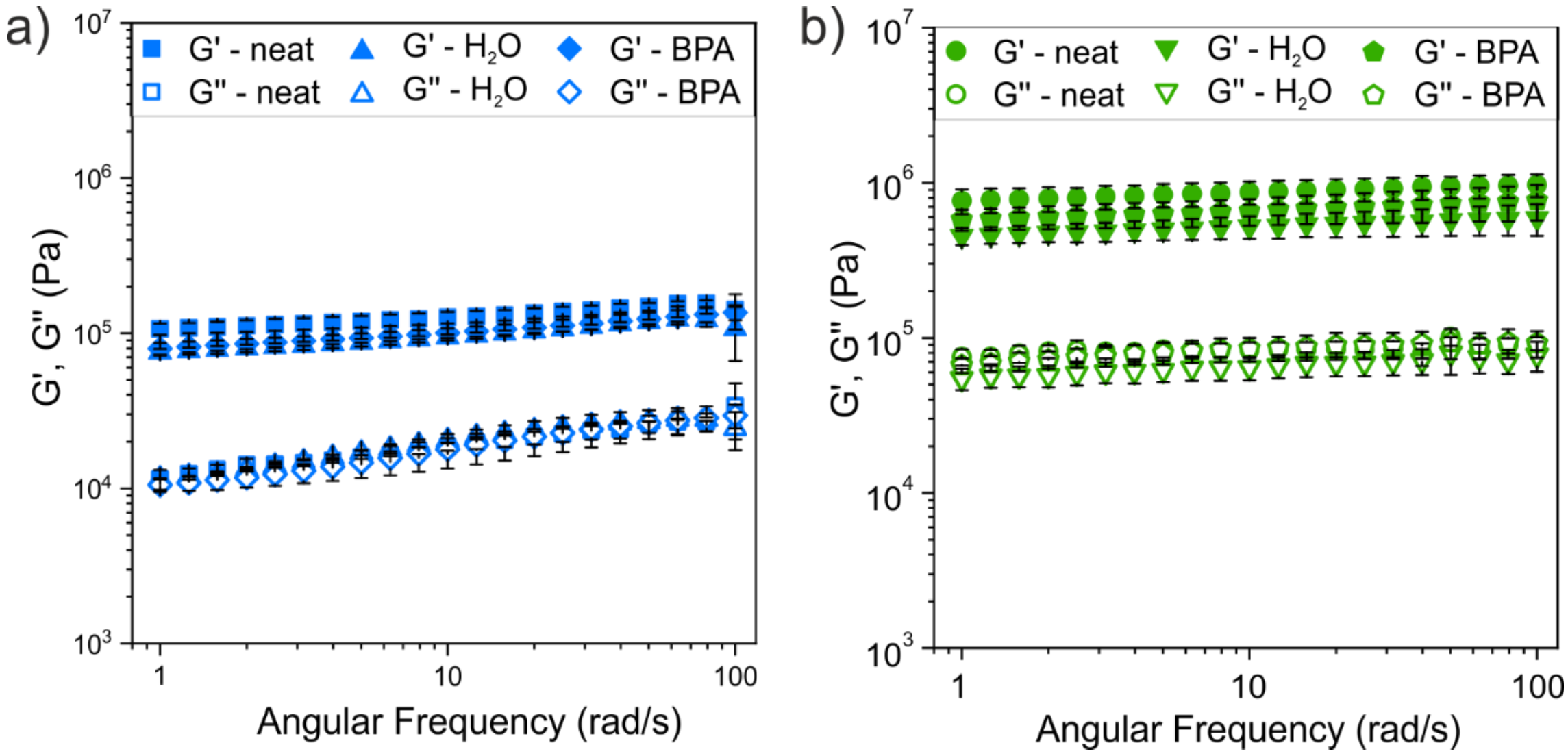

Figure S4. Angular frequency dependence of the storage modulus (G', filled markers) and loss modulus (G'', empty markers) measured at 25°C for TOPO:Men-DBS2 (a) and TOPO:Men-DBS5 (b) under different conditions: freshly prepared ("neat", blue squares in (a) and green circles in (b)), after contact with water ("H_2O", blue triangles in (a), green triangles in (b)), and after BPA uptake ("BPA", blue diamonds in (a), green pentagons in (b)). Error bars represent standard deviation across replicates. Where not visible, error bars are smaller than the marker size.

Results of SLE experiments

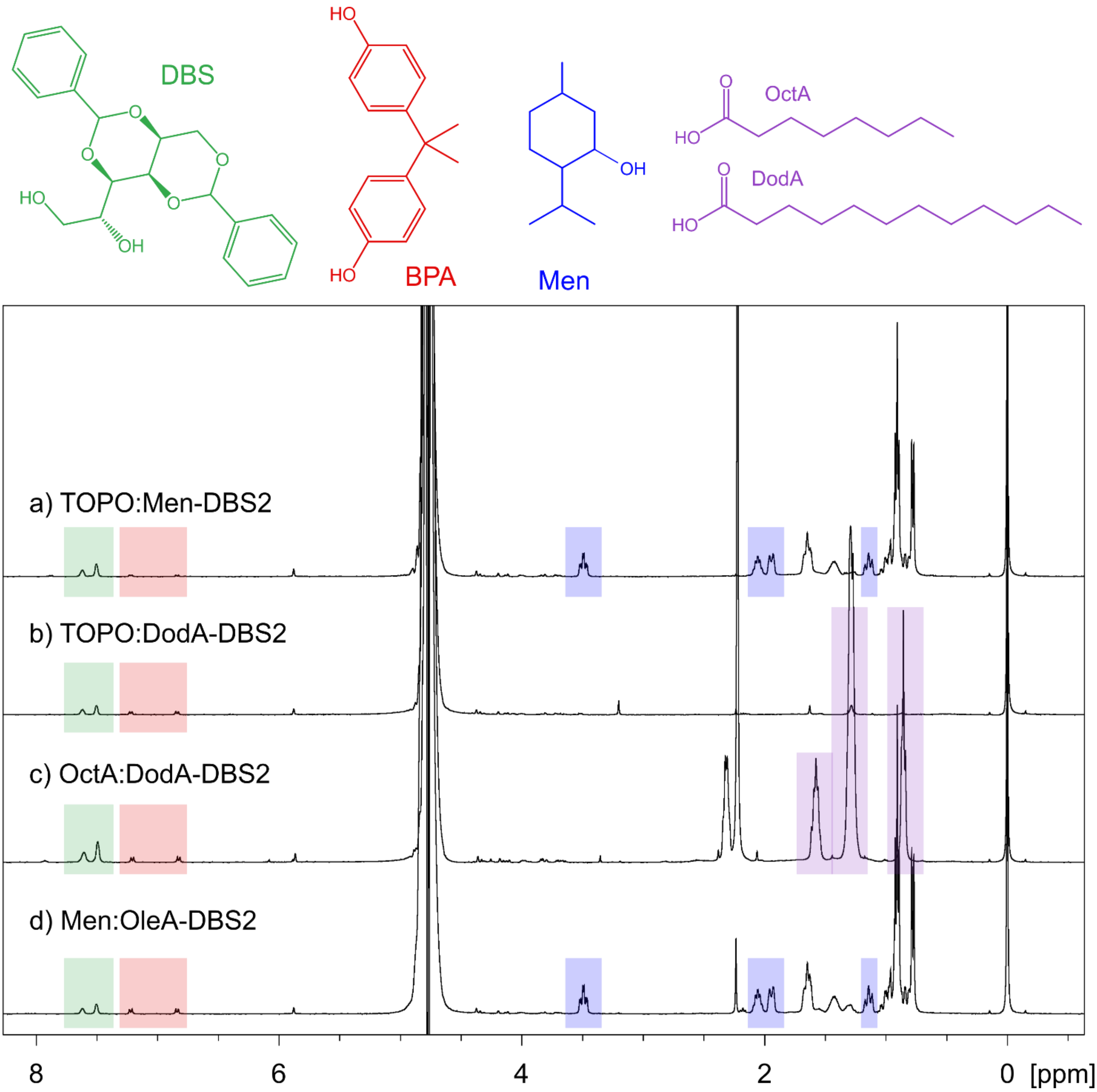

Figure S5. 1D ^{1}H liquid-state NMR spectra at 25 °C of the water phase after representative SLE experiments (stirring 1 h) with a) TOPO:Men-DBS2, b) TOPO:DodA-DBS2, c) OctA:DodA-DBS2, and d) Men:OleA-DBS2. The proton signals selected for qNMR quantification of the leaching of the individual species (DBS, BPA, Men and OctA/DodA).

Table S3. BPA extraction efficiency (EE), with relative standard deviation (rsd), and leaching of the Men component (Leach(Men)) and DBS (Leach(DBS)), using TOPO:Men-DBS2 and TOPO:Men-DBS5 in SLE under various experimental conditions, and comparison with TOPO:Men HES in LLE (from ref.[1]).

Extractant	Condition	EE [%]	rsd	Leach(Men) [%]	Leach(DBS) [%]
TOPO:Men-DBS2	1h – orbital shaker	59	15	0.11±0.01	0.28±0.04
TOPO:Men-DBS2	1h - stirring	97.3	0.4	0.23±0.03	0.75±0.04
TOPO:Men-DBS5	1h – orbital shaker	54	17	0.24±0.02	0.25±0.14
TOPO:Men-DBS5	1h - stirring	97.7	0.4	0.33±0.02	0.40±0.05
TOPO:Men-DBS5	16h – orbital shaker	>98.5	9	0.15±0.01	0.28±0.02
TOPO:Men	10min - stirring	>98.5	9	0.08±0.01	-

Table S4. BPA extraction efficiency (EE), with relative standard deviation (rsd), average leaching of the components (Leach(HES)) and leaching of DBS (Leach(DBS)), using TOPO:Dod-DBS2 and TOPO:Dod-DBS5 in SLE under various experimental conditions, and comparison with TOPO:DodA HES in LLE (from ref.[1]).

Extractant	Condition	EE [%]	rsd	Leach(HES) [%]	Leach(DBS) [%]
TOPO:DodA-DBS2	1h – orbital shaker	42	3	-	0.29±0.03
TOPO: DodA-DBS2	1h - stirring	94.2	0.8	-	0.61±0.08
TOPO: DodA-DBS5	1h – orbital shaker	74	24	-	0.21±0.09
TOPO: DodA-DBS5	1h - stirring	96.6	0.4	-	0.39±0.07
TOPO:DodA-DBS5	16h–orbital shaker	>98.5	9	-	0.38±0.07
TOPO:DodA	10min - stirring	>98.5	9	0.007±0.003	-

Table S5. BPA extraction efficiency (EE), with relative standard deviation (rsd), average leaching of the components (Leach(HES)) and leaching of DBS (Leach(DBS)), using OctA:DodA-DBS2 and OctA:DodA-DBS5 in SLE under various experimental conditions, and comparison with OctA:DodA HES in LLE (from ref.[1]).

Extractant	Condition	EE [%]	rsd	Leach(HES) [%]	Leach(DBS) [%]
OctA:DodA-DBS2	1h – orbital shaker	77	9	0.29±0.03	1.5±0.6
OctA:DodA-DBS2	1h - stirring	89	1	0.28±0.03	1.5±0.1
OctA:DodA-DBS5	1h – orbital shaker	91.9	0.4	0.31±0.04	1.8±0.3
OctA:DodA-DBS5	1h - stirring	84	3	0.27±0.03	1.2±0.1
OctA:DodA	10min - stirring	98.2	1.8	0.113±0.007	-

Table S6. BPA extraction efficiency (EE), with relative standard deviation (rsd), and leaching of the Men component (Leach(Men)) and DBS (Leach(DBS)), using Men:OleA-DBS2 and Men:OleA-DBS5 in SLE under various experimental conditions, and comparison with Men:OleA HES in LLE (from ref.[1]).

Extractant	Condition	EE [%]	rsd	Leach(Men) [%]	Leach(DBS) [%]
Men:OleA-DBS2	1h – orbital shaker	95.7	0.3	0.31±0.02	0.8±0.4
Men:OleA-DBS2	1h - stirring	92	4	0.32±0.01	0.6±0.1
Men:OleA-DBS5	1h – orbital shaker	92	1	0.34±0.03	0.8±0.2
Men:OleA-DBS5	1h - stirring	88	5	0.31±0.02	0.7±0.3
Men:OleA	10min - stirring	>98.5	9	0.13±0.03	-

Green metrics

Analytical EcoScale

An initial analysis was conducted using the Analytical EcoScale approach introduced by Gałuszka *et al.* as an extension of the previously established EcoScale concept,[2] where parameters related both to reagents and processes are considered. Although this metric was originally designed for assessing the sustainability of analytical methods in quantification processes, it is adapted here to the SLE protocol and the different HEG. To this end, we accounted for all steps, starting from HEG preparation to contaminant quantification.

For the reagent penalty points (PPs), calculations were based on the Global Harmonized System (GHS) pictograms and signal words as specified in the safety data sheets (SDS) of the actual supplier (Table S7). Each hazard pictogram corresponds to one PP. If a chemical is labeled with the signal word "Danger" (as for DodA and TOPO), the number of hazard pictograms is multiplied by two, whereas for chemicals labeled "Warning" (i.e. Men), the pictogram count remains unchanged. The total PPs for each binary mixture are determined by summing the PPs of both precursors (Table S8). Since the amount of reagents used in all cases is below 10 mL or 10 g, the final hazard PPs remain unadjusted (multiplied by 1).

For the instrument PPs, calculations took into account the actual energy consumption of each lab equipment (calculated considering the power in watts, the usage hours and the number of samples) during each step of the procedure. This includes using the hot plate for HES and HEG preparation (1 hour), the orbital shaker for extraction (16 hours), and NMR for quantification (Table S8). No additional PP was assigned for "Occupational Hazard" since no hazardous vapors or gases were emitted. The amount of waste is in the range 1-10 mL (3 PPs) and no treatment is performed (with a cost of 3 PPs).

Overall, both systems are ranked as excellent in the Analytical EcoScale metrics (score > 75), with the only difference related to the reagent hazard, which costs 3 PP for TOPO:Men and 4 PPs for TOPO:DodA.

Table S7. Penalty points (PP) introduced in the Analytical EcoScale [2] for the safety of the reagents.

	Number of pictograms	Symbol label	PPs
Men	1	warning	1
DodA	1	danger	2
TOPO	1	danger	2

Table S8. Analytical EcoScale [2] evaluation for the SLE protocol (16 hours orbital shaker and NMR quantification) using two TOPO-based HEG with DBS 5%.

Conditions			TOPO:Men-DBS5	TOPO:DodA-DBS5
**Reagents 1**	**Amount**	PPs		
	< 10 mL (g)	1	1	1
	10-100 mL (g)	2		
	> 100 mL	3		
**Reagent 2**	**Hazard (physical, environmental, health)**			
	None	0		
	less severe hazard	1	1	
	more severe hazard	2	2	2+2
**Reagent total PP**	**Amount PP x Hazard PP**		**3**	**4**
**Instrument 1**	**Energy**			
	≤ 0.1 kWh per sample	0		
	≤ 1.5 kWh per sample	1		
	> 1.5 kWh per sample	2	**2**	**2**
**Instrument 2**	**Occupational hazard**			
	Analytical process hermetization	0	**0**	**0**
	Emission of vapors and gases to the air	3		
**Instrument 3**	**Waste**			
	none	0		
	< 1 mL (g)	1		
	1-10 mL (g)	3	**3**	**3**
	> 10 mL (g)	5		
	recycling	0		
	degradation	1		
	passivation	2		
	no treatment	3	**3**	**3**
	Sum penalty points		14	15
	**Analytical EcoScale**		**86**	**85**

NMR measurements

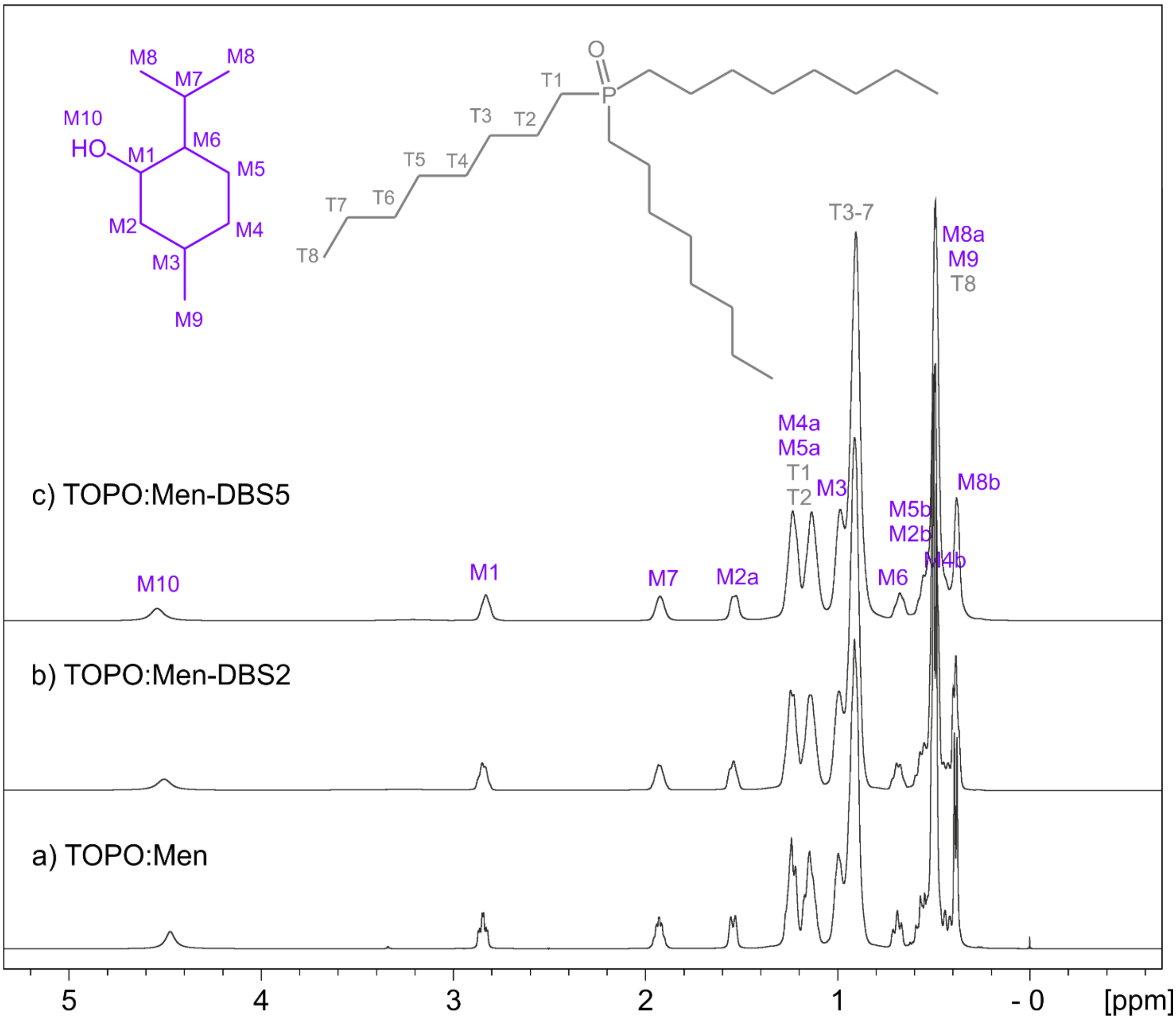

Figure S6. 1D ^{1}H liquid-state NMR spectrum at 25 °C of TOPO:Men (a) and comparison with 1D ^{1}H HRMAS NMR spectra at 25 °C of HEG TOPO:Men-DBS2 (b) and TOPO:Men-DBS5 (c).

Table S9. Apparent self-diffusion coefficients (in $m^2 \cdot s^{-1}$) of the components of HES and HEG (in $m^2 \cdot s^{-1}$) measured at 25 °C by ^{1}H PFG-NMR spectroscopy on the neat systems, after contact with water or BPA uptake.

Sample	$D_{Men\text{-}OH}$	$\Delta D_{Men\text{-}OH}$	D_{Men}	ΔD_{Men}	D_{TOPO}	ΔD_{TOPO}
TOPO:Men	2,47E-11		2,37E-11		1,32E-11	
TOPO:Men-DBS2	4,03E-11	2,95E-12	3,62E-11	7,08E-12	2,22E-11	4,03E-12
TOPO:Men-DBS2 + H_2O	7,97E-11	8,14E-12	7,07E-11	7,13E-12	6,19E-11	4,99E-12
TOPO:Men-DBS2 + BPA	4,98E-11	9,65E-12	4,41E-11	1,02E-11	2,16E-11	1,08E-12
TOPO:Men-DBS5	3,63E-11	9,50E-13	3,14E-11	1,44E-12	1,89E-11	5,25E-14
TOPO:Men-DBS5 + H_2O	6,21E-11	1,33E-11	3,99E-11	1,34E-11	2,28E-11	5,96E-12
TOPO:Men-DBS5 + BPA	3,62E-11	2,66E-12	2,35E-11	4,13E-12	1,65E-11	1,85E-12

References

[1] C. Carotti, G.I.C. Righetti, A. Mannu, A. Rossetti, L. Riva, G. Nicastro, F. Briatico Vangosa, C. Punta, A. Mele, M.E. Di Pietro, Hydrophobic Eutectic Solvents for Sustainable Bisphenol A Extraction from Water: Screening and Selection Based on Key Performance Criteria, ACS Omega. 10 (2025) 44662–44674. https://doi.org/10.1021/acsomega.5c07390.

[2] A. Gałuszka, Z.M. Migaszewski, P. Konieczka, J. Namieśnik, Analytical Eco-Scale for assessing the greenness of analytical procedures, TrAC - Trends Anal. Chem. 37 (2012) 61–72. https://doi.org/10.1016/j.trac.2012.03.013.
